# HDAC inhibitors in kidney development and disease

**DOI:** 10.1007/s00467-012-2320-8

**Published:** 2012-10-07

**Authors:** Lauren L. Brilli, Lisa M. Swanhart, Mark P. de Caestecker, Neil A. Hukriede

**Affiliations:** 1 Department of Developmental Biology, University of Pittsburgh, 3501 5th Ave., 5061 BST3, Pittsburgh, PA 15213 USA; 2 Department of Medicine, Division of Nephrology, Vanderbilt University Medical Center, Nashville, TN USA

**Keywords:** Histone deacetylase, HDACi, Kidney development, Kidney regeneration, Renal injury, Renal disease

## Abstract

The discovery that histone deacetylase inhibitors (HDACis) can attenuate acute kidney injury (AKI)-mediated damage and reduce fibrosis in kidney disease models has opened the possibility of utilizing HDACis as therapeutics for renal injury. Studies to date have made it abundantly clear that HDACi treatment results in a plethora of molecular changes, which are not always linked to histone acetylation, and that there is an essential need to understand the specific target(s) of any HDACi of interest. New lines of investigation are beginning to delve more deeply into target identification of specific HDACis and to address the relative toxicity of different HDACi classes. This review will focus on the utilization of HDACis during kidney organogenesis, injury, and disease, as well as on the development of these compounds as therapeutics.

## Introduction

Histone deacetylases (HDACs) play multiple roles during both kidney development [1] and the pathogenesis of kidney disease [2]. The continued development of isoform-selective histone deacetylase inhibitors (HDACis) will provide the scientific community with the necessary tools to study the individual roles that HDACs play during these processes. The goals of this review are to summarize what is currently known about the requirements for specific HDACs during renal development, and to address how the therapeutic applicability of HDACis has expanded beyond the field of cancer and now applies broadly to the field of kidney disease.

Acetylation and deacetylation of nucleosomal histone proteins serves as a post-translation modification that regulates transcriptional activity. This mechanism involves the interplay between the activity of two enzyme classes: (1) histone acetyltransferases (HATs), which promote an open chromatin configuration and transcriptional activation, and (2) HDACs, which generally promote chromatin condensation and transcriptional repression. Specifically, most HDACs remove acetyl groups from the ε-amino moiety of lysine residues located on N-terminal histone tails. This leaves the histone with a net positive charge, which strengthens its electrostatic interaction with the DNA phosphate backbone and results in transcriptional repression of associated genes.

As members of large multi-protein complexes, HDACs also target many non-histone proteins [3] and, in some instances, participate directly in gene activation [4, 5]. For example, HDACs positively regulate the oncogene c-Jun as well as the anti-apoptotic gene Bcl-2 [5]. Additionally, it has been demonstrated that HDACs are required for interferon-induced gene expression, which is critical for an antiviral immunological response [6]. Therefore, the function of HDACs, either as corepressors or coactivators, appears to be context-dependent.

## HDAC classes

To date, 18 mammalian HDAC proteins have been identified, and they are divided into four classes based on similarity to yeast orthologs [7]. Class I, II, and IV enzymes depend on zinc for catalytic activity and contain a highly conserved deacetylase domain [2, 8]. Class III enzymes, which are not the focus of this review, are termed sirtuins. They are structurally distinct from other HDAC classes since the catalytic activity of these enzymes depends on NAD+ rather than zinc and histones are not their primary substrate [2, 8]. Class I is comprised of HDACs 1, 2, 3, and 8. Class II is subdivided into class IIa, containing HDACs 4, 5, 7, and 9, and class IIb, which contains HDACs 6 and 10. HDAC 11 is the sole member of class IV because its catalytic domain resembles the actives sites of both class I and class II enzymes [8].

Understanding HDAC substrate specificity, as well as identifying strategies for developing novel isoform-selective HDACis, depends on thoroughly distinguishing between and within HDAC classes (for a more in-depth review, please see [2, 8, 9].) Class I HDACs are expressed ubiquitously, are localized to the nucleus, and interact with corepressor complexes to exert their function. HDAC1 and HDAC2 are recruited to the NuRD (nucleosome remodeling and deacetylation), Co-REST (corepressor for RE1 silencing transcription factor), and Sin3 corepressor complexes, whereas HDAC3 is most often recruited to the SMRT/N-CoR (silencing mediator for retinoid and thyroid receptors/nuclear receptor corepressor) complex [10]. HDAC8 does not appear to be recruited to corepressor complexes and is the only class I HDAC with intrinsic enzymatic activity [11].

Class II HDACs are thought to have tissue-specific roles, and are expressed in both the cytoplasm and the nucleus [12]. Class IIa enzymes contain a mutation in the catalytic site, resulting in a 1,000-fold reduction in activity [8]. Therefore alternative mechanisms, such as the recruitment of class I HDACs [8] or the interaction with corepressor complexes such as SMRT and N-CoR [10, 13], may be required to achieve activity. It has been proposed that this class of HDACs might act as “receptors” rather than enzymes, since they often bind acetylated lysine residues without actually performing the deacetylation reaction [14]. Additionally, a class IIb member, HDAC6 appears to primarily target α-tubulin which may be a result of its unique structure, consisting of two catalytic domains and a zinc finger [15].

## Available HDAC inhibitors

By preventing the removal of acetyl groups, HDAC inhibitors provide transcriptional activators and repressors with greater access to DNA resulting in altered gene expression patterns. In the 1970s, sodium butyrate, a natural fatty acid produced by bacteria, was one of the earliest HDAC inhibitors reported to alter proliferation and gene expression in cell culture [16]. Approximately 20 years later, the antifungal agents trapoxin and trichostatin A (TSA) were isolated from bacterial and fungal sources, respectively [13]. These compounds were first noted for their ability to induce cell cycle arrest and cancer cell differentiation. It was later appreciated that these effects were the result of histone modifications [13, 17].

**Fig. 1:**
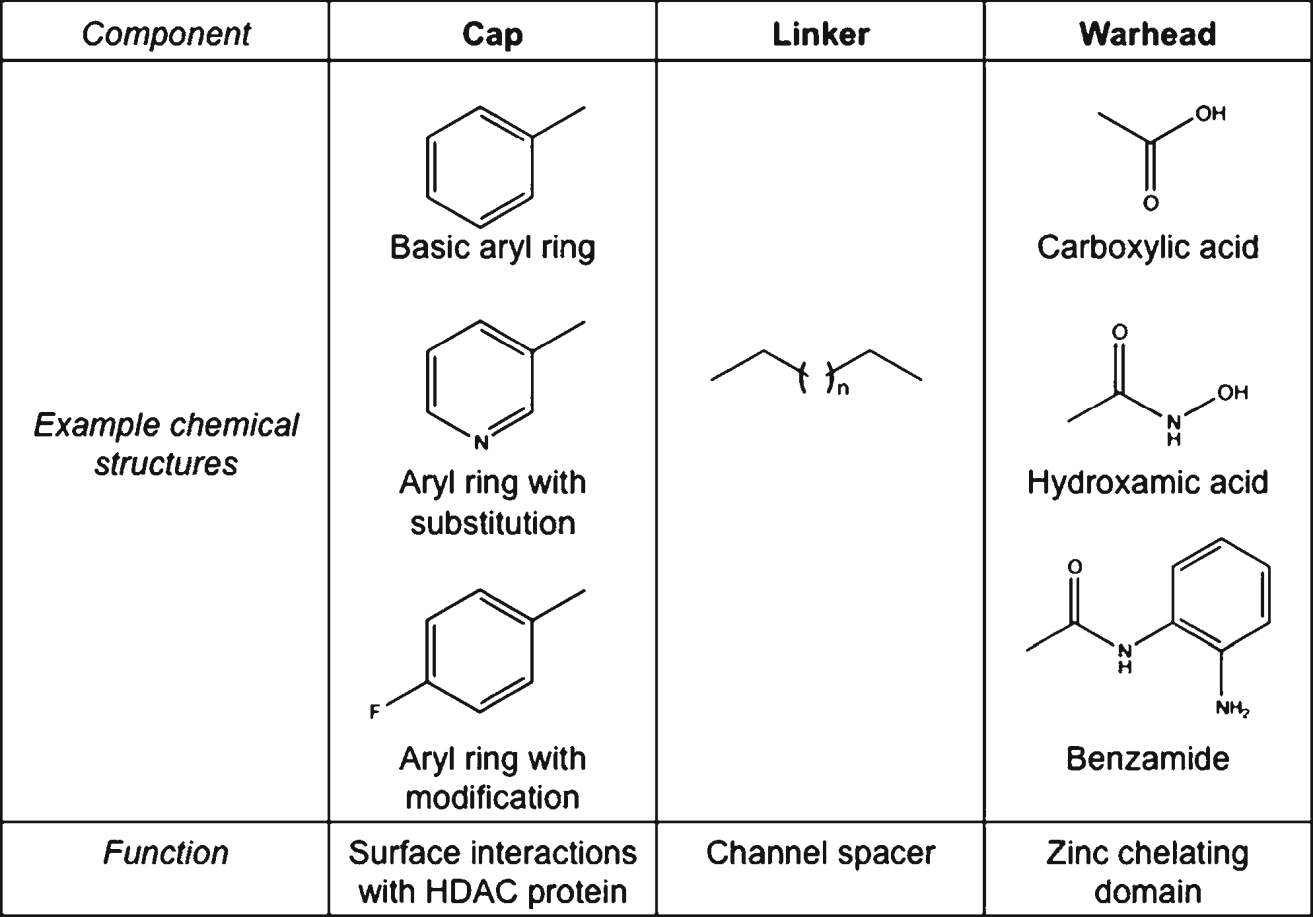
Characteristic design of histone deacetylases (HDAC) inhibitors. The general structure of HDAC inhibitors can be broken into three regions, each with a specific function: cap, linker, and warhead. Modifications can be introduced at each portion of the moiety, contributing to structural diversity

HDAC inhibitors targeting class I and II HDACs generally follow a classic warhead-linker-cap structure (Fig. 1). The warhead, or chelator, binds zinc in the HDAC catalytic site, rendering the enzyme inactive. The linker is a carbon chain that connects the warhead and cap and spans the length of the HDAC pocket. The cap, or surface-binding domain, interacts with residues on the surface of the HDAC enzyme and contributes to isoform specificity of the inhibitor [14]. There are at least four categories of HDACis that adhere to this structure: short chain fatty acids, hydroxamic acids, aminobenzamides and cyclic peptides. The short chain fatty acids are carboxylic acids that include valproic acid (VPA), butyric acid, phenyl butyrate (PBA), and 4-(phenylthio) butanoic acid (PTBA). These inhibitors are relatively weak and display some class I specificity. The hydroxamic acids are more broad-spectrum inhibitors capable of inhibiting class I and II HDACs, and include TSA, suberoylanilide hydroxamic (SAHA), LBH-589, LAQ-824, and PXD-101. Aminobenzamides are class I-selective and include SNDX-275, MGCD0103, and MS-275. Finally, there are several cyclic peptides, such as depsipeptide (romidepsin/FK-228) and apicidin, which appear moderately selective for class I HDACs [2, 17]. Although originally isolated as natural compounds, several synthetic analogs have now been created [18, 19].

The development of the first synthetic HDACi for therapeutic use in humans began somewhat serendipitously in the 1970s. It had been shown that dimethyl sulfoxide (DMSO) and other polar solvents could induce both differentiation and growth arrest in cultured cancer cells [20]. Hypothesizing that these molecules bound a metal ion in the HDAC active site led to the production of compounds such as SAHA (vorinostat), which possess a hydroxamic acid warhead and therefore chelate zinc more efficiently [21, 22]. As with TSA, SAHA inhibits class I and II HDACs, and was the first FDA-approved HDAC inhibitor for the treatment of refractory cutaneous T-cell lymphoma (CTCL) [17]. Due to their pro-differentiation, anti-proliferative effects, HDACis have generated excitement in the cancer field as novel chemotherapeutic agents [23, 24]. Since SAHA, about a dozen other compounds have entered clinical trials [14] (See Table 1) and, in 2009, the FDA also approved FK-228 (romidepsin), for treatment of CTCL [25]. Currently, SAHA and romidepsin are the only two FDA-approved HDAC inhibitors.

**Table 1:** Human clinical trials involving HDAC inhibitor therapies to treat renal cancer. Numerous HDACis have entered clinical trials as monotherapy and as combination therapy for the treatment of RCC. To date, only two HDACis have been approved by the Federal Food and Drug Administration, vorinostat and romidepsin. Keywords: Histone deacetylase, HDACi, kidney development, kidney regeneration, renal injury, and renal disease. For additional information on HDAC inhibitors in clinical trials in other cancer types, see clinicaltrials.gov or : www.fda.gov/ScienceResearch/SpecialTopics/RunningClinicalTrials/default.htm

HDACi class	Compound	Combination therapy	Clinical status	Major therapeutic target	References
Benzamides	Entinostat (MS-275)	Aldesleukin (Interleukin 2)	Phase I/II	Metastatic RCC	^a^
		13-*cis*-retinoic acid	Phase I	RCC and other solid tumors	[105]
Cyclic tetrapeptides	Romidepsin (FK228, depsipeptide)	Monotherapy	Phase II	Metastatic RCC	^a^, [113]
Hydroxamic acids	Vorinostat (SAHA)	Monotherapy	Phase II	Bladder and urethral cancer and advanced RCC	^a^
		Bevacizumab (VEGF inhibitor)	Phase I/II	Metastatic RCC	^a^
		13-*cis*-retinoic acid	Phase I/II	Advanced RCC	^a^
		SB-715992	Phase II	Advanced RCC	
		Ridaforolimus (mTOR inhibitor)	Phase I	Advanced RCC	^a^
		Sorafenib	Phase I	RCC and other solid tumors	^a^, [114]
	Panobinostat (LBH589)	Monotherapy	Phase II	Refractory Clear Cell RCC	^a^, [115]
		Sorafenib	Phase I	Advanced RCC	^a^
		Everolimus (mTOR inhibitor)	Phase I/II	Metastatic RCC	^a^
Short chain fatty acids	Valproic acid	Monotherapy	Phase I	Pediatric solid tumors	[116]

^a^clinicaltrials.gov

*HDAC* histone deacetylase, *RCC* renal cell carcinoma, *VEGF* vascular endothelial growth factor, *mTOR* mammalian target of rapamycin

## Toxicities and limitations of HDACi therapy

The HDACi literature suggests that compounds have differential and even paradoxical effects in cancer cells as compared to non-cancerous cells. Whereas HDAC inhibition is anti-proliferative in cancer cells, it can convey pro-proliferative signals in developmental settings [26]. In addition, HDACis are highly toxic to cancer cells yet appear to have cytoprotective effects in non-cancerous cells [2]. In fact, non-cancerous cells are much more resistant to high HDACi doses than cancer cells, and low doses have actually been shown to be both reno- [27, 28] and neuroprotective [29, 30]. One possible mechanism for differential sensitivity to SAHA treatment has been related to the availability of ROS scavenging proteins. SAHA treatment results in the upregulation of TBP-2 levels and a subsequent decrease in availability of ROS scavenging proteins. This effect, in combination with SAHA’s ability to cause increased ROS generation in cancer cells, may lead to preferential cancer cell death [17].

Despite apparent cytoprotective effects in some cell types, there is also evidence of cytotoxicity in non-cancerous cells following HDACi treatment. SAHA was shown to induce apoptosis in 35 % of a population of cultured rat renal proximal tubule cells [31]. In cultured mouse proximal tubule cells, TSA treatment resulted in the upregulation of the mitochondrial adapter protein p66sch. This increase is presumably linked to ROS generation since knockdown of p66sch attenuated ROS production in treated cells [32]. In general, hydroxamic acids, such as SAHA and TSA, are subject to modification via sulfation, which leads to the buildup of highly reactive, toxic sulfate metabolites of the hydroxy group [33]. For this reason, the therapeutic potential of TSA may be limited, even though it is widely used for research purposes. Based on these studies, it may be warranted to monitor renal function in patients undergoing HDACi therapy, particularly if the treatment regimen involves those classes found to have cytotoxic effects.

From a global perspective, patients tolerate HDACi therapy quite well, and the maximum tolerated dose has yet to be reached in some regimens [24]. Common side-effects of SAHA, romidepsin, and MS-275 include fatigue, nausea, and vomiting, although these are reversible upon treatment withdrawal [17, 24]. More worrisome, however, are the cardiac and immunologic effects, such as QT prolongation, thrombocytopenia, and/or myelosuppression, following HDACi treatment. Specifically, QT prolongation was observed during clinical trials with romidepsin, although confounding factors were also identified in specific patient populations [25] (See [24] for a more in-depth review of specific side-effects associated with individual HDAC inhibitors.) Additionally, valproic acid is a teratogen known to cause neural tube and other birth defects [34]. Although one study determined that TSA administered to pregnant mice did not harm either the mothers or the pups, further studies are warranted to examine the effects of HDACis during embryonic development [35].

One hypothesis for decreasing these toxicities includes the use of isoform-specific HDACis, rather than pan-inhibitors like SAHA and romidepsin [2]. To make this feasible, a viable, high-throughput assay testing isoform selectivity of novel compounds is necessary. Bradner et al. have developed an elegant kinetic assay for HDACs 1 through 9, which has been validated by profiling 20 known HDAC inhibitors currently being used in either research or clinical settings [14]. This provides both researchers and clinicians with valuable information about the precise isoform selectivity of compounds so that mechanistic and off-target effects can be evaluated.

## HDACs and HDACi in kidney development

Many laboratories have contributed to the current understanding of the roles of HDACs during embryonic development (for a good review see [9]). Initial studies have focused on knockout mice where the function of a single HDAC has been completely abrogated. Interestingly, these mice display a myriad of phenotypes. For ubiquitously expressed HDACs [8], such as HDAC1 and HDAC3, knockout results in early embryonic lethality [36–39]. For those HDACs that display tissue-specific expression, loss of function is generally more tolerated. For example, HDAC5 and HDAC9 knockout mice are both viable although they develop stress-induced myocardial hypertrophy [40, 41].

In addition to these more general requirements for HDACs during development, investigators are interested in understanding the role that these enzymes play in organogenesis. In the mouse, HDAC4 regulates the extent of chondrocyte hypertrophy, by inhibiting the activity of the runt-related transcription factor-2 (Runx2), and therefore is critical for skeletogenesis [42]. In the zebrafish, both Hdac1 and Hdac3 have been studied in detail. Interestingly, *hdac1* mutants and morphants proceed through early development without complication [43]. However, by 48 h post-fertilization (hpf), edema is apparent and circulation has been compromised. There is an absence of craniofacial cartilage structures as well as a reduction in the development of the pectoral fins. Retinal disorganization is also observed, and a requirement for Hdac1 in cell cycle exit and subsequent differentiation of retinal progenitors has been shown [43, 44]. Additionally, specification and differentiation of the liver and exocrine pancreas are severely delayed in *hdac1* mutants owing to reduced proliferation within the endoderm [45, 46]. Treatment of zebrafish embryos with non-teratogenic levels of valproic acid, a class I-specific HDACi, phenocopies *hdac1* mutants [46]. Interestingly, rescue of reduced liver size can be achieved by injection of *hdac3* mRNA as opposed to *hdac1* mRNA, suggesting an additional requirement for Hdac3 in zebrafish liver development.

Recently, data from both zebrafish and mice have implicated HDACs in the development of the pronephric and metanephric kidneys, respectively. In the zebrafish, specification of renal progenitor cells within the intermediate mesoderm occurs by 12 hpf. Over the course of the next 36 h, these progenitor cells epithelialize, the nephron is patterned, and a blood supply is delivered such that the pronephros becomes the functional larval kidney [47]. Treatment of zebrafish embryos, beginning at approximately 2 hpf, with the HDACis PTBA, PBA, or TSA results in an increase in renal progenitor cell number [26]. This increase is proliferation-dependent, since treatment with hydroxyurea and aphidicolin abolishes the ability of PTBA to expand the progenitor cell field. It is also dependent on functional retinoic acid (RA) signaling. Interestingly, ectopic activation of the RA pathway in both zebrafish and Xenopus results in the expansion of the kidney field [48, 49], and inhibition of HDACs lowers the threshold of RA required to activate transcription [50]. This expansion of the kidney field, following treatment with PTBA, persists throughout development and ultimately compromises kidney function due to renal progenitor cell hyperplasia [26], reminiscent of the teratogenic effects that have been described for HDACis in the developing invertebrate and vertebrate embryo [50]. Therefore, tight regulation of HDAC activity in the early zebrafish embryo is required for both the control of RA-dependent transcription and proliferation as well as for the development of a functional pronephros.

Metanephric kidney development, which is preceded by the development and degeneration of the pronephric and mesonephric kidneys, begins with an outgrowth, known as the ureteric bud (UB), from the caudal end of the Wolffian duct into the surrounding metanephric mesenchyme (MM) [51, 52]. Reciprocal interactions between the UB and MM result in branching and nephron induction, respectively. Branching occurs in a very stereotypical pattern and ultimately contributes to the collecting system, which includes the collecting ducts, renal pelvis, and ureter. At the tips of each branch, cells of the MM condense and begin their transition from a mesenchymal to an epithelial cell fate. They first form a renal vesicle, which is spherical in shape and attached at one end to the UB. Subsequently, single clefts are formed within this vesicle, giving rise to the comma-shaped and then S-shaped body. The S-shaped body is patterned along its proximal-distal axis such that the distal end fuses with the UB to form one continuous tubule and the proximal end develops into the glomerulus, following invasion by endothelial cells.

Although there is a significant amount of data describing the function of individual HDACs during organogenesis, little is known about their specific roles during metanephric kidney development. To address this deficiency, one group has characterized the expression patterns of all class I and II HDACs within the mouse kidney. HDACs 1-4, 7, and 9 appear to be temporally and to some extent spatially regulated such that expression levels are at their peak within the embryo and steadily decline as the mouse matures [1]. In E13.5 and 15.5 kidneys, HDAC1, and its binding partner HDAC2, localize to the undifferentiated and condensing MM as well as to the nephron progenitors of the comma- and S-shaped bodies [1, 53]. Interestingly, both are also found within the UB branches. While this localization pattern persists in postnatal mice, at a time when new nephrons are being formed, the levels of these HDACs are significantly reduced in more differentiated structures [1, 53]. A similar expression pattern is shared by HDAC3 but, in addition, this HDAC is also localized to the glomerular podocytes [1, 53]. To determine the general function of all class I and II HDACs during metanephric development, E13.5 kidneys are either cultured with Scriptaid, an inhibitor of class I and II HDACs, or its inactive analog Nullscript, and RNA isolated from these samples is subjected to genome-wide microarray analysis [1]. Many genes are deregulated following HDACi treatment, including those involved in Wnt/β-catenin, TGF-β/Smad, and PI3K-Akt signaling [1, 54]. Transcription factors that promote MM induction and survival, such as *Eya1*, *Pax2*, *WT1*, and *Emx2*, are among the most sensitive, showing reduced expression levels after only two hours of treatment [1]. This transcriptional deregulation has a significant impact on the development of the metanephros. Following Scriptaid treatment, kidneys appear hypoplastic with reduced UB branching, reduced proliferation, and increased apoptosis. Thus, high HDAC activity in the metanephric kidney is critical for promoting cell survival through transcriptional regulation. While these data demonstrate that HDACs are essential for kidney organogenesis, it does not address the differential roles that individual HDACs play during this process. To this end, work from another group suggests that the regulated expression of HDACs and their downstream targets is required for kidney development to proceed in a controlled manner. The renin-angiotensin system (RAS) promotes UB branching through the repression of *Sprouty*, an inhibitor of *GDNF/Ret* signaling [55]. Interestingly, treatment of E12.5 kidneys with exogenous Angiotensin-II, the principal growth factor of the RAS, results in increased levels of HDAC1, which promotes *Sprouty* expression [56]. Therefore, it appears that tight regulation of *Sprouty* levels, achieved by modulating HDAC1 activity, is required for proper branching of the ureteric bud during kidney morphogenesis. Taken together, these data highlight the importance of epigenetic regulation during metanephric kidney development.

## HDACi in kidney disease and injury

Whether it involves tubular epithelial cells that lead to cyst formation in polycystic kidney disease or glomerular epithelial and mesangial cells leading to glomerulosclerosis, the pathogenesis of many kidney diseases is characterized by dysregulation of cellular proliferation, leading to fibrosis. Since HDAC inhibition has been shown to be anti-fibrotic in the lung, liver, and skin [35], it should not be surprising that HDACis have been shown to have anti-fibrotic effects in models of acute and chronic kidney disease.

**Fig. 2:**
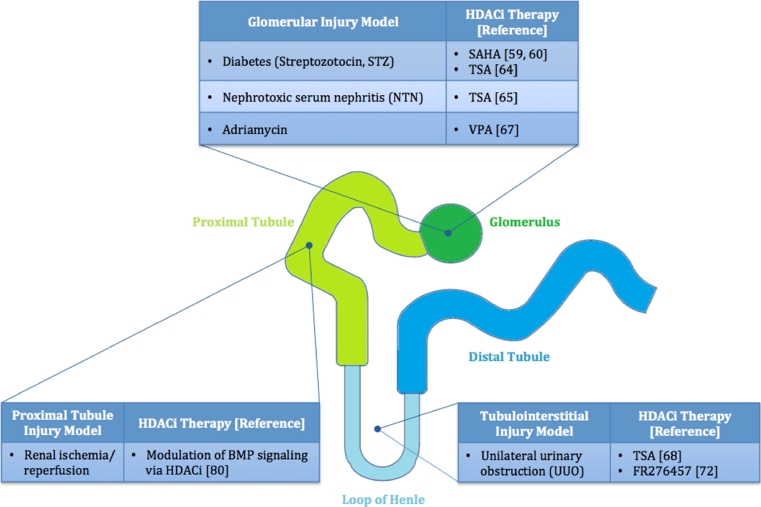
Summary of histone deacetylases (HDAC) inhibitor therapies used in renal injury models. Several HDACis have been found to attenuate fibrotic, inflammatory, and proliferative features of renal disease in mammalian models. The studies highlighted in this review are organized here by location of damage in the nephron in the injury model

HDACs were first implicated in the pathogenesis of non-cancerous kidney disease in 2003, when Mishra et al. demonstrated that TSA decreased proteinuria and the proliferative hallmarks of glomerulonephritis associated with SLE-induced lupus in mice [57]. Although this group was initially interested in the anti-inflammatory effects of HDACis, they recognized their anti-proliferative effects in some cell populations. Since 2003, HDACis have been investigated for their anti-fibrotic and anti-inflammatory effects in renal disease [2], and there is emerging evidence that HDACis may play a role in promoting tissue regeneration after acute kidney injury. We will discuss the anti-fibrotic and anti-inflammatory effects of HDACis in the context of various models of kidney disease, as well as their pro-regenerative effects following renal injury (summarized in Fig. 2).

## Glomerular disease

### Diabetic nephropathy

Although often clinically silent, renal hypertrophy (enlarged kidney) is one of the earliest features of diabetic nephropathy. This may precede podocyte loss that leads to proteinuria. Renal hypertrophy is associated with glomerular hyper-filtration, which is thought to be an underlying mechanism promoting progressive renal injury in diabetic nephropathy. Although the underlying mechanisms promoting renal hypertrophy are complex, there is evidence that this results from activation of mammalian target of rapamycin (mTOR) signaling in the diabetic kidney [58]. Two studies have demonstrated that SAHA can attenuate renal hypertrophy in rodent models of streptozotocin-induced diabetes [59, 60]. Advani et al. found that SAHA treatment decreased mouse kidney size after 18 weeks compared to controls [59], and Gilbert et al. similarly demonstrated that a 4-week SAHA course resulted in attenuation of renal hypertrophy in rats [60]. While the mechanism by which HDACi reduces renal hypertrophy in this model is unknown, this group observed a decrease in apoptosis in SAHA-treated kidneys, indicating that HDACi treatment does not reduce kidney size via cellular toxicity.

Diabetic nephropathy is also characterized by glomerular sclerosis resulting from excess deposition of matrix in the glomerular mesangium, and loss of glomerular epithelial cells, followed by aberrant fibrosis within the glomerular structure. There is a substantial body of evidence to suggest that this mechanism is mediated by activation of TGF-β signaling in the diabetic glomerulus [61–63]. TGF-β1 has been shown to upregulate HDAC2 in rodent models of diabetes, and treatment with nonselective or class I-selective HDACis prevents fibrosis [64]. Additionally, HDAC2 knockdown in cell culture decreased the accumulation of extracellular matrix components, further implicating HDAC2 in fibrosis. Thus, class I inhibition, and in particular HDAC2 inhibition, seems to have therapeutic potential in attenuating the fibrotic features of diabetic nephropathy. Finally, oxidative stress is also thought to play a role in regulating diabetic nephropathy fibrosis [56]. Noh et al. observed that H_2_O_2_, a potent inducer of oxidative stress, stimulated an increase in HDAC2 levels [64]. SAHA treatment, however, decreased markers of oxidative damage in the kidneys of diabetic mice [59]. These studies indicate a key role for HDACis in attenuating oxidative damage and fibrosis in diabetic nephropathy.

### Glomerulonephritis

HDACi treatment has shown promise in several models of glomerulonephritis. Nephrotoxic serum nephritis (NTN) is a rat model characterized by the development of an aggressive, proliferative, crescentic glomerulonephritis giving rise to progressive glomerulosclerosis. It is therefore a model of rapidly progressive glomerulonephritis in humans. TSA ameliorates proliferative glomerulonephritis, long-term glomerulosclerosis, and proteinuria in this model [65]. Adriamycin induces a less proliferative glomerular disease in rats, characterized by glomerular epithelial cell injury and progressive glomerulosclerosis, and is a model of human focal segmental glomerulosclerosis (FSGS) [66]. Pre-treating mice with VPA, a class I-selective HDACi, before adriamycin injection ameliorated proteinuria as well as sclerotic disease features, and preserved most features of podocyte morphology [67]. Additionally, a course of VPA treatment after adriamycin injection was able to improve proteinuria and sclerosis compared to untreated controls. VPA treatment reduced both proliferation in the glomerulus, as measured by Ki67, and the expression of fibrotic markers, including TGF-β1. This is consistent with data from diabetic nephropathy models that implicate HDAC inhibition in the prevention of fibrosis via TGF-β1 suppression. Thus, class I HDAC suppression seems to have a therapeutic role in attenuating fibrosis in models of both diabetic nephropathy and FSGS.

## Tubular and interstitial diseases

### Tubulointerstitial fibrosis

Tubulointerstitial fibrosis is the common hallmark of chronic kidney diseases from a variety of different causes. It is characterized by renal tubular atrophy and progressive expansion of the tubulointerstitial space with fibroblasts and by the abnormal deposition of extracellular matrix components, including type 1 collagen. The pathogenic mechanisms of tubulointerstitial fibrosis are diverse and complex. However, key elements include: 1) activation of TGF-β signaling in fibroblasts, promoting fibroblast proliferation and deposition of abnormal extracellular matrix; and 2) infiltration with inflammatory cells which promote tissue injury and fibrosis by releasing inflammatory cytokines and reactive oxygen species. While therapeutic options in the clinic are limited, HDACi is a promising treatment for targeting both the pro-fibrotic and inflammatory pathogenic aspects of this condition [61, 63].

To prevent renal fibrosis, researchers have focused their efforts on elucidating the mechanisms of TGF-β signaling. TSA treatment prevents TGF-β1-dependent responses in cultured human renal tubule epithelial cells (RTECs) [57]. Cells treated with TSA still demonstrated Smad protein phosphorylation, which relays TGF-β1 signaling to the nucleus, indicating that TSA blocks TGF-β1’s effects downstream of these factors. Co-treatment of human RTECs with TGF-β1 and TSA prevented TGF-β-induced apoptosis by blocking caspase protein cleavage (also observed in an in vivo model [68]). This effect was mediated by inhibiting extracellular signal-regulated kenase (ERK) activation. This latter finding is notable since recent studies have shown that the epidermal growth factor receptor (EGFR), is required for sustained TGF-β-dependent fibrosis in a mouse model of angiotensin II-induced renal fibrosis [69]. Thus, this work proposes a dual therapeutic role for HDAC inhibition in preventing tubular interstitial fibrosis: (1) suppressing the pro-fibrotic effects of TGF-β1, and (2) ameliorating renal tubular atrophy by protecting against tubular epithelial cell apoptosis.

Additional mechanisms have been proposed for attenuating tubular damage-mediated fibrosis through HDAC inhibition. Pang et al. induced tubulointerstitial injury in mice via unilateral urinary obstruction (UUO), and correlated a decrease in fibrotic markers in TSA-treated kidneys [68]. This was associated with decreased levels of phosphorylated STAT3. Since STAT3 has been implicated in the regulation of tubulointerstitial fibrosis following UUO [70] and is a known HDAC target, these findings suggest HDACi may also decrease renal fibrosis by decreasing STAT3 expression and signaling in this model of chronic kidney disease. Thus, HDACis are likely contributing in multiple ways to exert anti-fibrotic effects.

HDACis have also been implicated in the prevention of inflammatory infiltration after renal damage. Following UUO, there is an increase in both HDAC1 and HDAC2 in the tubular epithelium. While treatment with TSA decreased fibrosis scores, it also decreased macrophage infiltration and expression of CSF-1, a secreted cytokine that promotes proliferation and survival of monocyte/macrophages. Similarly, TSA and VPA were able to attenuate TNF-α-induced CSF-1 secretion by cultured renal tubular epithelial cells [71]. Work by another group demonstrated that treatment with FR276457, a hydroxamic acid derivative that inhibits both class I and II HDACs, after UUO resulted in decreased MCP-1 levels, a chemotactic cytokine that normally attracts macrophages to the kidney shortly after UUO [72]. However, the role of macrophages in acute and chronic kidney injury is complex. It involves both the detrimental effects of early, inflammatory M1 macrophages, and late, anti-inflammatory M2 macrophages that also enhance epithelial regenerative responses [73]. Therefore HDACis likely decrease pro-inflammatory M1 macrophages early, and/or promote expansion of anti-inflammatory and pro-regenerative M2 macrophages later in the course of disease.

### Cystic disorders

Polycystic kidney disease (PKD) is characterized by the development of fluid-filled cysts, lined by epithelial cells, in the renal parenchyma. In humans, autosomal dominant PKD is caused primarily by mutations in PKD1 or PKD2 [12, 74]. These genes encode polycystins, proteins that coordinate calcium flux in association with primary cilia via mechanisms not well understood [75]. This coordination may serve as a sensor for epithelial proliferation since mutations in either gene lead to excessive proliferation and cyst formation [12, 74, 75]. Currently, therapies are limited and the majority of PKD progresses to end stage renal disease [74, 76, 77]. While most therapeutic strategies aim to inhibit either mTOR signaling or the renin-angiotensin system, no single-drug regimen has been identified that successfully inhibits cyst formation [77]. As the mechanisms of cyst formation in PKD pathogenesis are beginning to unfold, HDACs have emerged as a novel therapeutic target.

Using zebrafish as a model system, chemical screens identified TSA as a compound that could partially suppress phenotypes correlated with cyst formation in larval PKD models. Both TSA and VPA were able to retard cyst formation in *pkd2* mutant zebrafish larvae, and knockdown of *hdac1* in larvae suppressed the *pkd2* mutant phenotype. Interestingly, these observations were translatable to a mammalian system. While VPA treatment in *Pkd1* knockout mice slightly decreased cyst formation [74], TSA reduced cyst formation in *Pkd1* mutant embryos [76]. These data point to the potential for class I HDACis in the further study of PKD etiology.

Mechanistically, p53 deacetylation may be the link between HDACs and cyst formation. Not only is p53 a direct target of HDAC1 [12], but it can also bind the promoter of PKD1, resulting in transcriptional repression [78]. Interestingly, inhibiting both class I and II HDACs with TSA alleviated PKD1 transcriptional repression in vitro [78]. Further experiments are warranted to determine specifically whether class I or class II HDACs are involved in this transcriptional regulation since there is also evidence implicating class II HDACs in cyst formation. Using an in vitro assay with polycystin 1 mutant cells, it was shown that HDAC5 is phosphorylated and exported from the nucleus in response to shear stress, allowing it to activate downstream targets [75]. Furthermore, it was demonstrated that cystic kidney phenotypes were reduced in *Hdac5*, *Pkd2* double null mouse embryos, and that TSA administration decreased cyst formation in *Pkd2* mutant embryos.

An alternative mechanism for HDACi attenuation of cyst formation involves cell cycle regulation via p21 and Rb-E2F1 signaling. *Pkd1* mutant cells enter S phase more frequently than wild-type cells, leading to enhanced proliferation and cyst formation. TSA treatment decreased S-phase entry in these cells in vitro through a mechanism involving reduced Id2 levels and increased p21 levels. This work implicates HDAC inhibition in cell cycle regulation as a mechanism for decreasing epithelial proliferation and attenuating cyst formation [76, 79].

## HDACi in kidney injury

In addition to anti-fibrotic and anti-inflammatory effects, there is also evidence that epigenetic modulation through HDACis can promote regeneration after tissue injury by reactivating the expression of signaling machinery normally required during kidney organogenesis. This “regeneration recapitulates development” paradigm underlies the regenerative capacity of several mammalian organs, and we are only beginning to understand the extent to which this mechanism plays a role in kidney regeneration.

Marumo et al. [80] observed that exposure to transient renal ischemia resulted in histone hypoacetylation in mouse proximal tubule epithelial cells. This led them to investigate whether HDACs played a role in modulating the kidney’s response to ischemic injury. In an in vitro model, recovery after reperfusion was associated with decreased levels of HDAC5, and knockdown of HDACs 1, 2, or 5 induced expression of BMP7. In vivo, fewer HDAC5-positive cells were observed in proximal tubules after reperfusion, which correlated with histone hyperacetylation and induction of BMP7 expression.

These experiments suggest that proximal tubule recovery following renal ischemia may be modulated by HDAC activity, perhaps through the reactivation of BMP7. While BMP7 is expressed in the developing mammalian kidney, it is absent in the proximal tubules of the adult kidney. Since BMP signaling is reactivated post-renal ischemia [81], and exogenous BMP7 treatment is therapeutic in rodent kidney injury models [82–84], the “regeneration recapitulates development” paradigm characteristic of other organs may also apply to the kidney. Importantly, these changes in histone acetylation status and gene expression occur during the recovery phase after reperfusion, implying that treatment with an HDACi may be a useful therapy *after injury has occurred* in a hospital setting.

Accordingly, treatment with TSA prevented proteinuria and BUN elevation in NTN mice [65]. This global attenuation of renal injury was associated with activation of BMP7 expression in an uncharacterized side population of cells identified by flow cytometry. Although controversial, some believe this side population may constitute a population of intrarenal stem cells [85, 86]. Since TSA treatment did not increase BMP7 expression in other renal cells, this population may serve as a source of BMP7 to promote renal regeneration. This suggests that pharmaceutical HDACi therapy may have the potential to stimulate the BMP pathway, or other developmental pathways, and promote regeneration, thus ameliorating kidney injury.

## HDAC inhibitors and renal carcinoma

The critical role that HDACs play in cancer progression has been appreciated for some time, since changes in HDAC expression or inappropriate recruitment of these enzymes has been observed in a number of human cancers [17]. Class I HDACs, specifically HDACs 1 and 2, are highly expressed in renal cell carcinoma (RCC), making these particularly interesting therapeutic targets [87]. Because of this, researchers have focused much of their efforts on designing effective treatments to inhibit HDACs. This is clearly evident by the number of treatments that are currently in clinical trials (Table 1) [88]. The mechanisms of action vary for these inhibitors, although the identities of the “key” targets are still not completely clear [89]. Regardless, HDACis have been shown to induce growth arrest, apoptosis, and differentiation in a number of tumor cell lines as well as in mouse tumor models. They are also important for inhibiting tumor angiogenesis, which is normally critical for growth and progression of solid tumors [90, 91].

In RCC, HDACis play an important role in promoting both cell cycle arrest and apoptosis. Treatment of RCC cell lines with VPA inhibits proliferation and results in increased levels of p21, a cyclin-dependent kinase inhibitor [92]. While HDACis have been touted for their ability to promote G_1_ arrest, primarily through the up-regulation of p21 [17], these small molecules are also capable of inducing arrest at the G_2_-M transition [89]. This G_2_-M arrest has been documented in RCC cell lines treated with the HDACi LBH589 (Panobinostat) as well as the novel γ–lactam-based HDACi KBH-A145 [93, 94]. Mechanistically, LBH589 treatment reduces the protein levels of both Aurora A and B kinases, which normally play important roles during mitosis, and this effect is mediated specifically through the inhibition of HDAC3 and HDAC6 [93]. Since the levels of both Aurora A and B kinases are significantly higher in tumors taken from RCC patients, the use of HDACis as potential therapies for RCC is certainly supported.

Currently, it appears that combinatorial therapy involving HDACis holds the most promise for treating RCC (Table 1), and there have been a number of studies utilizing this approach. First, combinatorial therapy enhances anti-proliferative effects. VPA, in combination with low-dosed interferon-α, is more effective at reducing overall HDAC activity as well as inhibiting cell proliferation in RCC cell lines compared with single-agent treatments [92]. Additionally, this combinatorial effect is seen when VPA is used in conjunction with AEE788, a receptor tyrosine kinase inhibitor, or RAD001, an inhibitor of mTOR [95, 96]. Likewise, the combination of SAHA with either the protease inhibitor ritonavir or the topoisomerase I inhibitor topotecan results in more potent inhibition of HDACs, promotion of pRb dephosphorylation, and reduced proliferation [97, 98].

Second, combination therapy involving HDACis appears to attenuate the levels of hypoxia inducible factors (HIFs) which are often dysregulated in clear cell RCC due to the inactivation of the tumor suppressor gene, von Hippel-Lindau (VHL) [99]. This effect can be augmented when HDACis, particularly LBH589, are used in conjunction with rapamycin, an inhibitor of the mammalian target of rapamycin [100]. Since HIFs are pro-angiogenic transcription factors that support tumor growth by promoting vascularization, these studies not only implicate HDAC involvement in the upregulation of HIFs during tumor growth, but they also suggest a role for HDACis in RCC treatment.

Finally, HDACis have been used in combination with retinoic acid (RA) to promote cancer cell differentiation, which is compromised during cancer development and serves as a hallmark of the disease [89]. RA is a diffusible factor derived from vitamin A that has been used for the treatment of a number of cancers. However, some renal malignancies have shown resistance to this therapy [101, 102]. Data from RCC cell lines suggest that this retinoid-resistance is due to loss of *RARβ2* expression [103]. Treatment with MS-275 (Entinostat) results in hyperacetylation of the *RARβ2* promoter and re-expression of this gene, restoring sensitivity of these lines to retinoids. When MS-275 is combined with 13-*cis*-retinoic acid (cRA), there is a significant inhibition of RCC tumor growth in mouse xenografts. Similar results have been obtained with TSA and all-*trans* retinoic acid [104]. This combinatorial treatment of HDACi and cRA has been used in phase I clinical trials for patients with advanced solid tumors, including renal malignancies [105]. Taken together, these data suggest the HDAC inhibition coupled with additional therapies may be the most effective method of treatment for RCC.

## Towards the future

Since HDACis ameliorate AKI-mediated damage, promote regenerative responses, and decrease scarring in kidney disease models, the use of these compounds as renal injury therapeutics is now in the realm of possibility. However, the mechanism underlying how HDACis affect renal tubular epithelial cells during regeneration is unclear. In a post-damage environment, regenerating proximal tubular epithelial cells proliferate and express genetic markers normally associated with the early embryonic renal epithelia (in the pre-tubular aggregates and renal vesicles). These markers, which include Pax2, Wnt4, Lhx1 and components of the Notch signaling pathway [81, 106–108], normally appear within the first 24 h following injury and are subsequently lost as the cells undergo epithelial differentiation. Possibly, by promoting a more open chromatin environment, HDACis may contribute to the re-expression of these embryonic markers and thus regeneration, although the exact mechanism regulating embryonic gene reactivation following kidney damage is currently unknown. While work to date provides correlative evidence, a direct link between HDACi treatment and embryonic gene reactivation has yet to be established. As mentioned in this review, recent studies have demonstrated a reversible reduction in HDAC activity and increased histone acetylation (K9 acetyl-histone H3) in regenerating proximal tubular epithelial cells following renal ischemia [80]. As epigenetic regulation of gene expression during development is in part regulated by histone acetylation [8], these findings suggest that alterations in HDAC activity could mediate the epigenetic reprogramming of regenerating tubular cells to a more primitive embryonic epithelial state. Interestingly, work in an iPS reprogramming study demonstrated that VPA could drive mouse embryonic fibroblasts to a more primitive, embryonic stem cell-like state [109]. Therefore, understanding how HDACis influence the transcriptional profile of renal tubular epithelial cells during regeneration is essential to understanding how these compounds can be used as AKI therapeutics.

To successfully develop new therapeutic HDACis, an appreciation for the balance between potency and toxicity as well as the myriad of substrate targets is required. Moreover, while preliminary studies indicate that these agents have beneficial effects in diverse disease processes (Fig. 2), the mechanisms by which they mediate these effects need to be precisely defined in order to improve drug design and targeting. Therefore, simply designing the most effective pan-HDACi, that works at the lowest concentration, will likely result in a poor candidate for drug development. Similarly, pursuing the “nanomolar rule” could either result in rejecting effective compounds, which could have been the case with SAHA [17], or selecting those that are highly toxic and produce off-target effects in animal models [110]. Likely, the most pressing hurdle to overcome in drug development is assessing the toxicity associated with certain categories of HDACis. The hydroxamic acid class of HDACis has been shown to be relatively toxic in the kidney. SAHA induces apoptosis in 35 % of a population of cultured rat renal proximal tubule cells [31], and in cultured mouse proximal tubule cells, TSA treatment results in the upregulation of targets linked to ROS generation [32]. In general, hydroxamic acids are subject to sulfation, which leads to the buildup of highly reactive, toxic sulfate metabolites of the hydroxy group [33]. Although TSA is widely used for research purposes, its therapeutic potential may be limited and, based on these studies, it may be warranted to monitor renal function in patients undergoing hydroxamic acid HDACi therapy. Decreasing associated HDACi toxicities may likely include the use of class-specific HDACis, rather than pan-inhibitors like SAHA and romidepsin [2]. However, data to support this claim is currently limited, and there is evidence that a class I inhibitor, MS-275, shows a similar toxicity profile in cancer patients to the more broad-spectrum inhibitors [111]. Therefore, developing the ultimate therapeutic HDACi may require a combination of class specificity and non-toxic metabolic products.

Additionally, the development of novel HDACis depends on a thorough understanding of the molecular mechanism of action for each compound, including the interaction between the HDACi and the target HDAC as well as the specific response this interaction creates [112]. This will allow for the effective design of drugs that are capable of distinguishing within and between HDAC classes. As described above, progress has been made in this pursuit since it is now appreciated that HDACs function as both corepressors and coactivators, and these effects appear to be context-dependent. These enzymes are capable of targeting many non-histone proteins and, in some instances, participating directly in gene activation [4, 5]. Therefore, the molecular mechanism of action can vary for individual inhibitors which is likely due to the fact that the identity of the main target(s) is still not completely clear [89]. Defining these targets will hopefully guide the development and effective application of isoform-selective HDACis. The prowess of HDACis as successful therapeutics in a wide array of clinical applications, including the treatment of cancer, has been clearly demonstrated. Thus, the stage has been set for their future use in the treatment of AKI.
